# Obtusifolin improves cisplatin-induced hepatonephrotoxicity via the Nrf2/HO-1 signaling pathway

**DOI:** 10.1007/s00210-025-03900-x

**Published:** 2025-02-20

**Authors:** Selcan Cesur, Berrin Yalinbas-Kaya, Ali Tureyen, Fahriye Zemheri-Navruz, Hasan Huseyin Demirel, Sinan Ince

**Affiliations:** 1 Department of Gastroenterology, Ministry of Health Eskisehir City Hospital, 26080 Eskisehir, Turkey; 2 Department of Molecular Biology and Genetics, Faculty of Science, Bartın University, 74100 Bartın, Turkey; 3 Bayat Vocational School, Afyon Kocatepe University, 03780 Afyonkarahisar, Turkey; 4 Department of Pharmacology and Toxicology, Faculty of Veterinary Medicine, Afyon Kocatepe University, 03200 Afyonkarahisar, Turkey

**Keywords:** Cisplatin-induced hepatonephrotoxicity, Obtusifolin, Oxidative stress, Inflammation, Nrf2/HO-1

## Abstract

Cisplatin (CIS) is a highly effective chemotherapeutic drug, but one of its most serious side effects is hepatonephrotoxicity, which varies based on its dosage and duration of use. Previous studies have reported that obtusifolin (OBS) exhibits several pharmacological effects, including antioxidant and antidiabetic activities. In this study, we investigated the protective effects of OBS against CIS-induced hepatonephrotoxicity. OBS (0.5 and 1 mg/kg, i.p.) was administered to male mice for 10 days, while CIS (20 mg/kg, i.p.) was administered on day 7 to induce hepatonephrotoxicity. The results showed that OBS reduced the CIS-induced elevations in AST, ALT, ALP, BUN, and creatinine levels by approximately 14%, 11%, 9%, 18%, and 14%, respectively. OBS also decreased liver and kidney MDA levels by approximately 31% and 25%, while enhancing liver and kidney GSH, SOD, and CAT levels by 50–36%, 80–70%, and 95–55%, respectively. In association with oxidative stress and the apoptotic process, OBS reduced liver and kidney mRNA expression levels of Nrf2 (by approximately 1.7- and 1.6-fold, respectively), HO-1 (by 1.6- and 1.4-fold, respectively), and Bcl-2 (by 1.6- and 1.4-fold, respectively). Additionally, OBS suppressed the mRNA expression levels of NF-κB (by 0.7- and 0.7-fold), TNF-α (by 0.6- and 0.6-fold), Bax (by 0.8- and 0.7-fold), and Cas-3 (by 0.7- and 0.7-fold). Protein expression analysis revealed that OBS increased Nrf2 (showing a 1.7- to 1.2-fold) and Bcl-2 levels (by 1.3- to 1.8-fold), and reduced Bax (by 0.7- to 0.8-fold) and caspase-3 (by 0.7- and 0.7-fold) levels altered by CIS treatment. Histopathological examinations confirmed that OBS reduced liver and kidney damage caused by CIS. In conclusion, OBS significantly improved CIS-induced hepatonephrotoxicity by reducing oxidative stress, inflammation, and apoptosis via modulation of the Nrf2/HO-1 pathway. These findings suggest that OBS could be a potential therapeutic agent for mitigating the side effects of chemotherapeutics.

**Supplementary Information:**

The online version contains supplementary material available at 10.1007/s00210-025-03900-x.

## Introduction

Cisplatin (CIS), a platinum-based chemotherapeutic drug, is widely used to treat various types of cancer. but it is associated with several adverse effects, including nausea, myelosuppression, vomiting, nephrotoxicity, ototoxicity, and hepatotoxicity (Habib et al. 2021). CIS exerts its anticancer effects primarily by forming DNA adducts, leading to cell cycle arrest and ultimately triggering apoptosis (Pabla et al. 2008). Among the most serious complications of CIS treatment, particularly at high doses, are liver and kidney damage (Wang et al. 2023). CIS toxicity is predominantly linked to the excessive production of reactive oxygen species (ROS), resulting in oxidative stress and inflammation (Elhady et al. 2022). Additionally, increased lipid peroxidation (LPO) and DNA damage compromise cellular integrity by affecting Bax and Bcl-2 levels, which in turn promote apoptosis (El-Sayed et al. 2019). As a result, various antioxidant compounds are being investigated to prevent CIS toxicity while maintaining its antineoplastic efficacy.

The nuclear factor erythroid 2-related factor 2 (Nrf2) plays a critical role in regulating oxidative stress (Ince et al. 2024). Under normal conditions, Nrf2 is sequestered in the cytoplasm by Keap1; however, under stress, it activates multiple antioxidant enzymes (such as HO-1, GPx, CAT, and SOD) and inflammatory mediators, including NF-κB (Tureyen et al. 2023). In CIS-induced toxicity, the oxidative stress-induced imbalance and subsequent release of Nrf2 are considered key factors contributing to organ damage (Xiang et al. 2022). Therefore, mitigating or treating CIS-induced liver and kidney toxicity necessitates the use of drugs or compounds capable of counteracting oxidative stress.

Obtusifolin (OBS), an anthraquinone compound found in the seeds of *Cassia obtusifolia*, a plant commonly used in traditional Chinese medicine, has demonstrated a range of pharmacological effects, including analgesic (He et al. 2014), antioxidant (Zhuang et al. 2016), antidiabetic (Ali et al. 2021), anti-metastatic in breast cancer (Hsu et al. 2014), and cartilage protective properties in osteoarthritis (Nam et al. 2021). Several studies have shown that OBS suppresses mitochondrial apoptosis triggered by high glucose uptake in human umbilical vein endothelial cells and decreases capillary cell apoptosis in the retinas of diabetic rats (Hou et al. 2014). Additionally, OBS has been reported to significantly improve scopolamine-induced neurological disorders in mice (Kim et al. 2009) and reduce hyperlipidemia and hyperglycemia in diabetic rats, effects attributed to its antioxidant properties (Tang and Zhong 2014). However, the potential effects of OBS against CIS toxicity have not yet been explored. This study examined the impact of OBS on CIS-induced hepatonephrotoxicity in mice, which arises from oxidative stress, using biochemical, oxidative, and molecular analyses.

## Material and methods

### Chemicals

CIS as an injectable form (Cipintu-100 mg/100 ml) was sourced from Koçsel Pharmaceuticals (Kocaeli, Turkey), and OBS was obtained from ChemFaces (CAS number: 477–85-0, Wuhan, Hubei, China). All other chemicals utilized in the research were acquired from commercial suppliers.

### Experimental animals and protocol

**Fig. 1:**
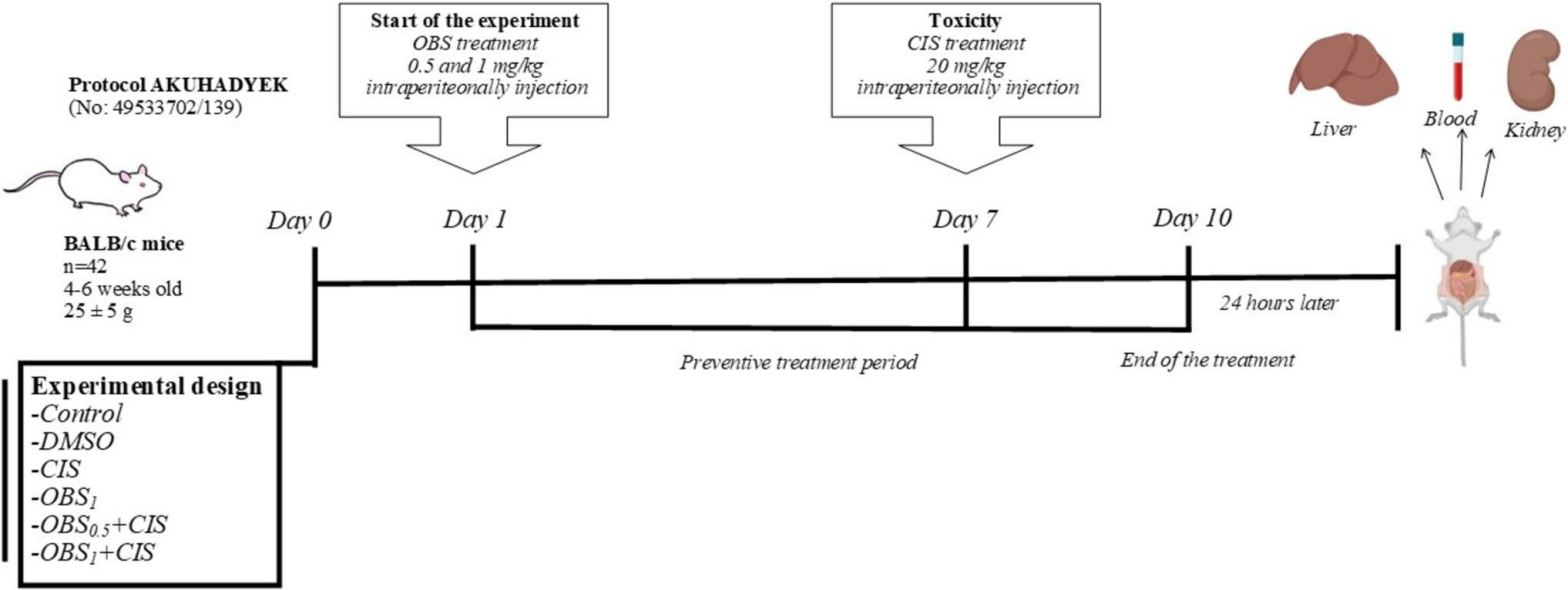
Experimental flowchart depicting the administration of obtusifolin (OBS) and cisplatin (CIS). OBS was administered intraperitoneally to mice at doses of 0.5 and 1 mg/kg daily for 10 days. CIS was administered intraperitoneally as a single dose of 20 mg/kg on the 7th day, and the experimental phase concluded at the end of the 10th day. AKUHADYEK: Afyon Kocatepe University local ethics committee, n: number of animals

Forty-two male BALB/c mice, 4–6 weeks old and weighing 25 ± 5 g, were acquired from the Experimental Animal Research and Application Unit at Afyon Kocatepe University. The study adhered to universal ethical guidelines, and approval was secured by the local ethics committee (AKUHADYEK-49533702/139). The mice were sustained in a standard surrounding environment, having a temperature of 21 ± 3 °C, humid conditions levels of 55–60%, and a 12-h light/dark cycle. All procedures followed the ARRIVE guidelines and the Guide for the Care and Use of Laboratory Animals as per the US National Institutes of Health. The mice were randomly divided into six groups, each containing seven animals, and were acclimatized for 7 days. The control group provided standard rodent chow and received 0.1 ml of physiological saline intraperitoneally throughout the experiment. The DMSO group was administered 0.1 ml of 1% DMSO intraperitoneally for 10 days. The CIS group received a single dose of 20 mg/kg CIS intraperitoneally on the 7th day. The OBS group was treated with 1 mg/kg OBS dissolved in DMSO, administered intraperitoneally for 10 consecutive days. In the OBS and CIS combination groups, 0.5 and 1 mg/kg OBS were administered intraperitoneally for 10 days, with a single dose of 20 mg/kg CIS given 1 h after OBS administration on the 7th day (Fig. 1). The dosing regimens for OBS (Kim et al. 2009) and CIS (Folayan et al. 2022; Wang et al. 2023) were adapted from prior studies. Twenty-four hours after the final treatment, the mice were anesthetized with an intraperitoneal injection of ketamine (84 mg/kg) and xylazine (11.2 mg/kg). Blood samples were collected via intracardiac puncture, and liver and kidney tissues were harvested. Plasma was separated from the collected blood, and portions of the kidney and liver tissues were homogenized in 0.15 M Tris–HCl buffer (pH 7.4) for biochemical analyses. For molecular studies, tissue samples were cryopreserved in liquid nitrogen and stored at − 80 °C until further use. Histopathological specimens were fixed in 10% formaldehyde and processed for examination.

### Biochemical parameters

The activities of alanine aminotransferase (ALT; LOT:200,005), aspartate aminotransferase (AST; LOT:200,003), and alkaline phosphatase (ALP; LOT:190,006), along with urea (BUN; LOT:16,015) and creatinine (LOT:101524B) levels in mouse plasma, were measured using commercial assay kits from BIOLABO (Medica, Germany). All assays were performed using a UV–VIS spectrophotometer (Shimadzu-1601, Tokyo, Japan) for spectrophotometric analysis.

### Lipid peroxidation and antioxidant enzyme activity measurements

Malondialdehyde (MDA) levels, as an indicator of LPO, and glutathione (GSH) concentrations in the tissue levels were measured following the protocols of Ohkawa et al. (1979) and Draper and Hadley (1990), respectively. Additionally, antioxidant enzyme activities, including superoxide dismutase (SOD), and catalase (CAT) were evaluated in tissue samples according to the procedures described by Sinha (1972) and Sun et al. (1988), correspondingly. Protein concentrations in tissue samples were measured by applying the method of Lowry et al. (1951). All analyses were performed using spectrophotometric techniques.

### Assessment of mRNA levels

Total RNA was extracted from kidney and liver tissues samples of mice using the RNA Purification Kit (Gene Jet, Thermo Fisher Scientific, USA). To eliminate any residual DNA, the extracted RNA was treated with DNase I (RNase-free, 1 U/μL, Thermo Fisher Scientific, USA). The purified RNA was then reverse transcribed into cDNA using the cDNA Reverse Transcription Kit (Thermo Fisher Scientific, USA). Target genes were amplified using specific primers, as listed in Table 1 (In supplementary material). Primers specific to Mus musculus were designed for this study using FastPCR 6.0 software (Kalendar et al. 2009), based on mRNA sequences for β-actin (housekeeping gene), Nrf2, HO-1, TNF-α, NFκB, Bcl-2, Cas-3, and Bax obtained from the NCBI database. For RT-qPCR, 1 μl of cDNA was combined with Master Mix Green (RealQ Plus 2x, Ampliqon, Denmark) and gene-specific primers, and the reactions were performed on RT-qCR system (CFX96 Bio-Rad, USA). The Amplification specificity was confirmed via melt-curve analysis, and data were collected using the system’s software. All samples were checked against β-actin, and results were stated as fold changes in cycle threshold (Ct) values relative to control samples, calculated using the 2^−ΔΔCt^ method (Pfaffl 2001).

**Table 1:** Primer sequences for mRNAs used in Real-time PCR

Gene name	GenBank accession number	Primer sequences (5′−3′)	Amplicon length (bp)
Nrf2	AH006764.2	F: TTCCATAGGACAATCACTCA R: ACACATGGCTATAAAATGCC	90
HO-1	NM_010442.2	F: GAAATCATCCCTTGCACGCC R: TTTGAACTTGGTGGGGCTGT	231
NFκB	AY521463.1	F: AGAGAAGGAGATCATCCGCCA R: AACACGGAAGCTGGCTTTGTA	416
TNF-α	NM_001278601.1	F: CCAACCAGGCAGGTTCTGTCC R: GGCTCTTGACGGCAGAGAGGA	554
Cas-3	XM_017312543.3	F: GTCATCTCGCTCTGGTACGG R: AGTCAGACTCCGGCAGTAGT	274
Bax	NM_007527.4	F: CACCTGAGCTGACCTTGGAG R: GAGGAAGTCCAGTGTCCAGC	308
Bcl-2	NM_009741.5	F: AAGCAGACGTAGAAGTGGGC R: AAGCAGACGTAGAAGTGGGC	201
β-actin	NM_007393.5	F: CGGGCTGTATTCCCCTCCATCG R: AGCACAGCCTGGATGGCTACG	338

### Western blotting

For the blotting analysis, liver and kidney tissues were homogenized in 1 × RIPA buffer with protease inhibitory properties The protein amounts were designated using the Qubit™ Protein (BR) Assay Kit (Catalog No: Q33211) with the Qubit Buffer Mix. The sample extracts (50 μg) were loaded onto a gel (Bolt™ 4–12% Bis–Tris Plus) and electrophoretically separated, followed by transfer onto a membrane. Antibody binding steps were performed using the Western System (iBind Flex, Thermo Fisher Scientific, Inc.). To minimize nonspecific binding, the membranes were incubated at 24 °C for 10 min in iBind Flex Solution, which consisted of iBind Flex buffer, additives, and distilled water. The primary antibodies (Nrf2, 1:1000, FNab05855, FineTest; Cas-3, 1:1000, E-AB-66940, Elabscience) and the secondary antibody (HRP Goat Anti-Rabbit, Catalog No: AS014, ABclonal) were applied as regards the manufacturer’s guidelines, for 2.5 h with incubation at room temperature. β-actin (AC026, Abclonal) was used as a loading control. Reactive bands were detected using chemiluminescence (Perkin Elmer, USA), and band intensities were analyzed using the ImageJ software.

### Immunohistochemical staining

Samples were initially processed with xylene and a graded alcohol series. To inhibit endogenous peroxidase activity, a 1% H_2_O_2_ solution was engaged for 30 min at 25 °C. After rinsing with distilled water for 2–3 min, the sections were immersed in citrate buffer and heated for 10 min. Following washes with PBS and distilled water, a UV block was applied for 10 min. Primary antibodies for Bax (1:100, sc7480, Santa Cruz) and Bcl-2 (1:300, sc7382, Santa Cruz) were applied, and the slides were incubated at 4 °C for 12 h. After washing with PBS, a secondary antibody (100 µl, HRP Goat Anti-Mouse IgG, Cat: AS003, ABclonal) was applied for 10 min at room temperature. Detection was carried out using streptavidin and chromogen (Cat. No. K3467; Dako; Agilent Technologies, Inc.), followed by counterstaining Mayer’s hematoxylin for 30 s. The slides were examined under a light microscope at × 200 magnification.

### Histopathological examination

Formaldehyde-fixed tissue samples were processed for paraffin embedding, and 5 μm-thick sections were prepared. These sections were stained with hematoxylin and eosin (H&E) following the protocol described by Tureyen et al. (2023). The stained slides were examined under a light microscope (Nikon, Tokyo, Japan), and images were captured using a Nikon camera (Japan). Tissue damage was scored as follows: 0, no damage; 1, mild damage; 2, moderate damage; and 3, severe damage. Statistical analyses were performed to compare group differences based on these scores.

### Statistical evaluation

The study data were analyzed using GraphPad Prism 8.0 software. Normality of data distribution was assessed first. For normally distributed data, one-way ANOVA was performed, followed by Tukey’s post hoc test to identify group differences. Results are expressed as mean ± standard deviation, with statistical significance set at *p* < 0.05.

## Results

### OBS enhances liver and kidney function parameters affected by CIS treatment

**Fig. 2:**
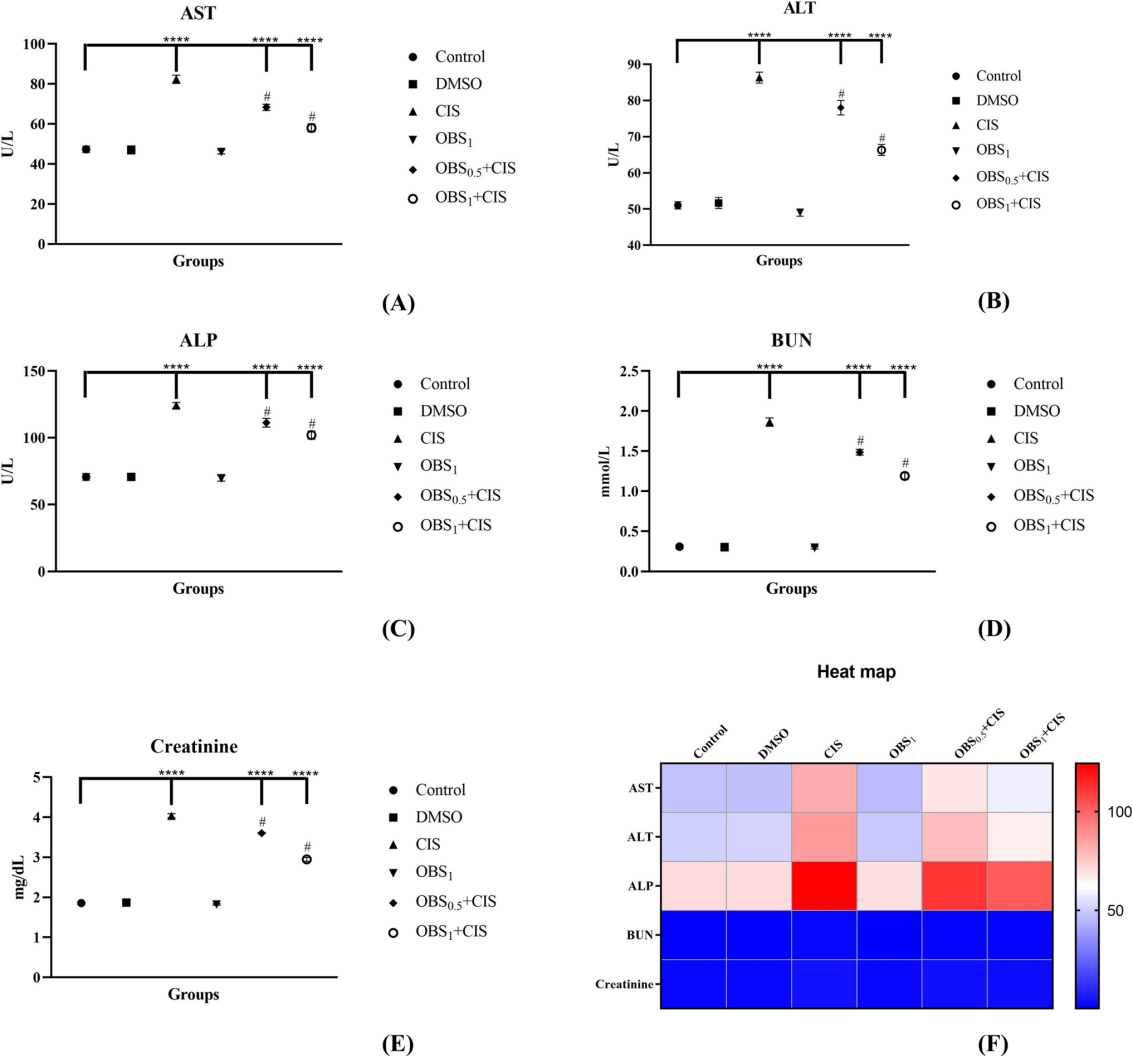
Effects of obtusifolin (OBS) and cisplatin (CIS) treatments on liver function parameters AST (**A**), ALT (**B**), and ALP (**C**) activities, as well as renal function parameters BUN (**D**) and creatinine (**E**) levels, with corresponding two-dimensional color tone representations (**F**). Groups: control, DMSO (dimethylsulfoxide, 1%), CIS (20 mg/kg), OBS_1_ (1 mg/kg), OBS_0.5_ + CIS (0.5 mg/kg OBS + 20 mg/kg CIS), and OBS_1_ + CIS (1 mg/kg OBS + 20 mg/kg CIS). Data are presented as mean ± standard deviation; *n* = 7. Values marked with different symbols (*) are statistically significant compared to the control group, while values marked with different symbols (#) are statistically significant compared to the CIS group (*p* < 0.0001)

Hepatic function parameters, including AST, ALT, and ALP, as well as renal function parameters, such as BUN and creatinine, were significantly elevated in the CIS-treated group (Fig. 2) compared to the control group (*p* < 0.0001). However, OBS administration markedly reduced these elevated levels caused by CIS, with average reductions of 14%, 11%, 9%, 18%, and 14%, respectively (*p* < 0.0001). Furthermore, treatment with OBS alone did not produce any significant alterations in these parameters compared to the control group (*p* > 0.05).

### OBS reduces CIS-induced lipid peroxidation and enhances antioxidant enzyme activities

**Fig. 3:**
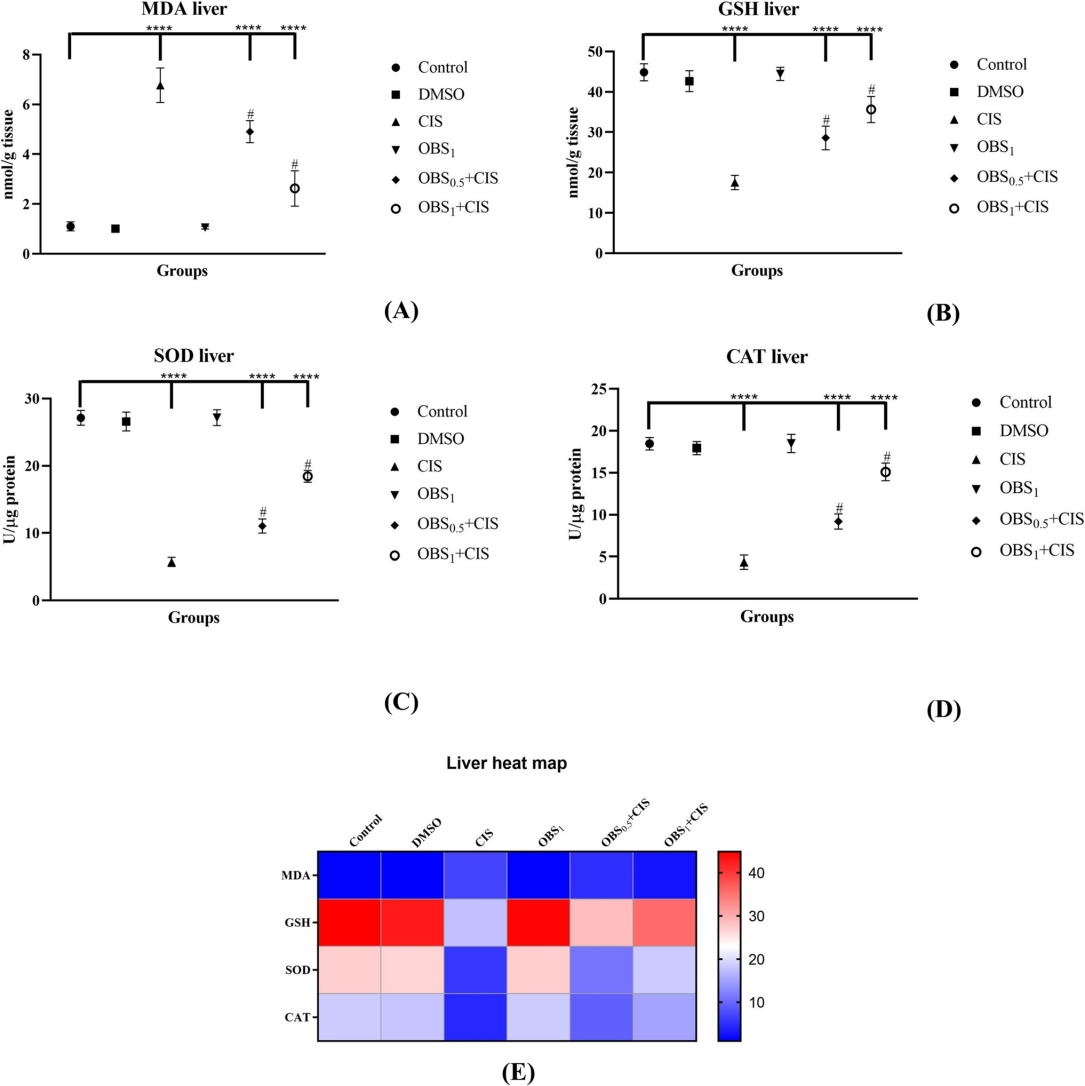
Effects of obtusifolin (OBS) and cisplatin (CIS) treatments on liver MDA (**A**) and GSH (**B**) levels and SOD (**C**) and CAT (**D**) activities and the with corresponding two-dimensional color tone representations (**E**). Groups: control, DMSO (dimethylsulfoxide, 1%), CIS (20 mg/kg), OBS_1_ (1 mg/kg), OBS_0.5_ + CIS (0.5 mg/kg OBS + 20 mg/kg CIS), and OBS_1_ + CIS (1 mg/kg OBS + 20 mg/kg CIS). Data are presented as mean ± standard deviation; *n* = 7. Values marked with different symbols (*) are statistically significant compared to the control group, while values marked with different symbols (#) are statistically significant compared to the CIS group (*p* < 0.0001)

**Fig. 4:**
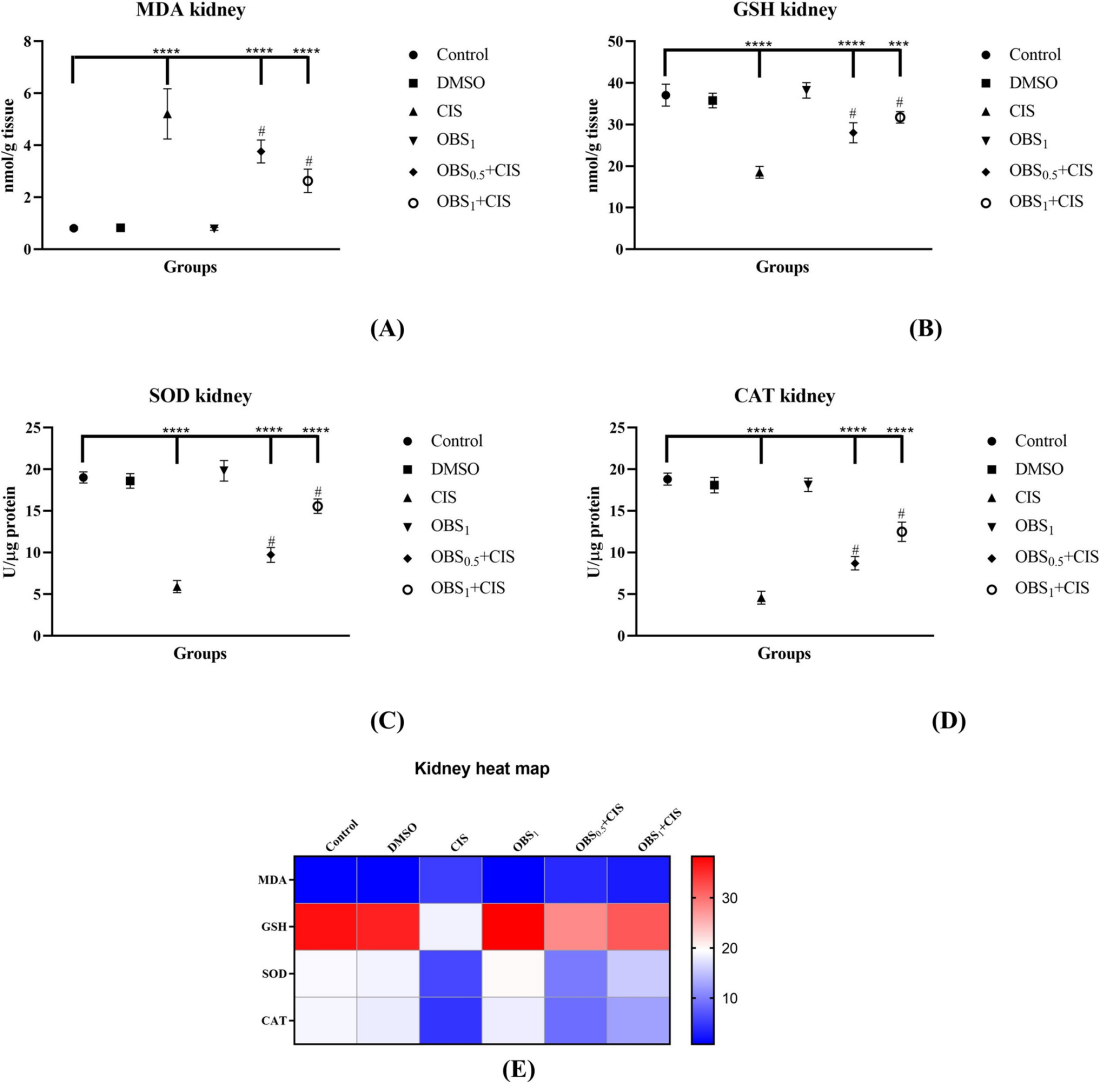
Effects of obtusifolin (OBS) and cisplatin (CIS) treatments on kidney MDA (**A**) and GSH (**B**) levels, as well as SOD (**C**) and CAT (**D**) activities, with corresponding two-dimensional color tone representations (**E**). Groups: control, DMSO (dimethylsulfoxide, 1%), CIS (20 mg/kg), OBS_1_ (1 mg/kg), OBS_0.5_ + CIS (0.5 mg/kg OBS + 20 mg/kg CIS), and OBS_1_ + CIS (1 mg/kg OBS + 20 mg/kg CIS). Data are presented as mean ± standard deviation; *n* = 7. Values marked with different symbols (*) are statistically significant compared to the control group, while values marked with different symbols (#) are statistically significant compared to the CIS group (*p* < 0.0001)

CIS treatment significantly increased MDA levels in both liver (Fig. 3) and kidney (Fig. 4) tissues compared to the control group, while GSH levels, along with SOD and CAT activities, were substantially decreased (*p* < 0.0001). However, OBS administration effectively reversed these CIS-induced changes, restoring the values closer to those of the control group (*p* < 0.0001). Specifically, OBS reduced MDA levels in the liver and kidney by 31% and 25%, respectively, while enhancing GSH, SOD, and CAT levels by 50–36%, 80–70%, and 95–55%, respectively. Furthermore, treatment with OBS alone did not result in any significant changes in these parameters compared to the control group (*p* > 0.05).

### OBS ameliorates mRNA and protein expression increased by CIS by the Nrf2/HO-1 pathway

**Fig. 5:**
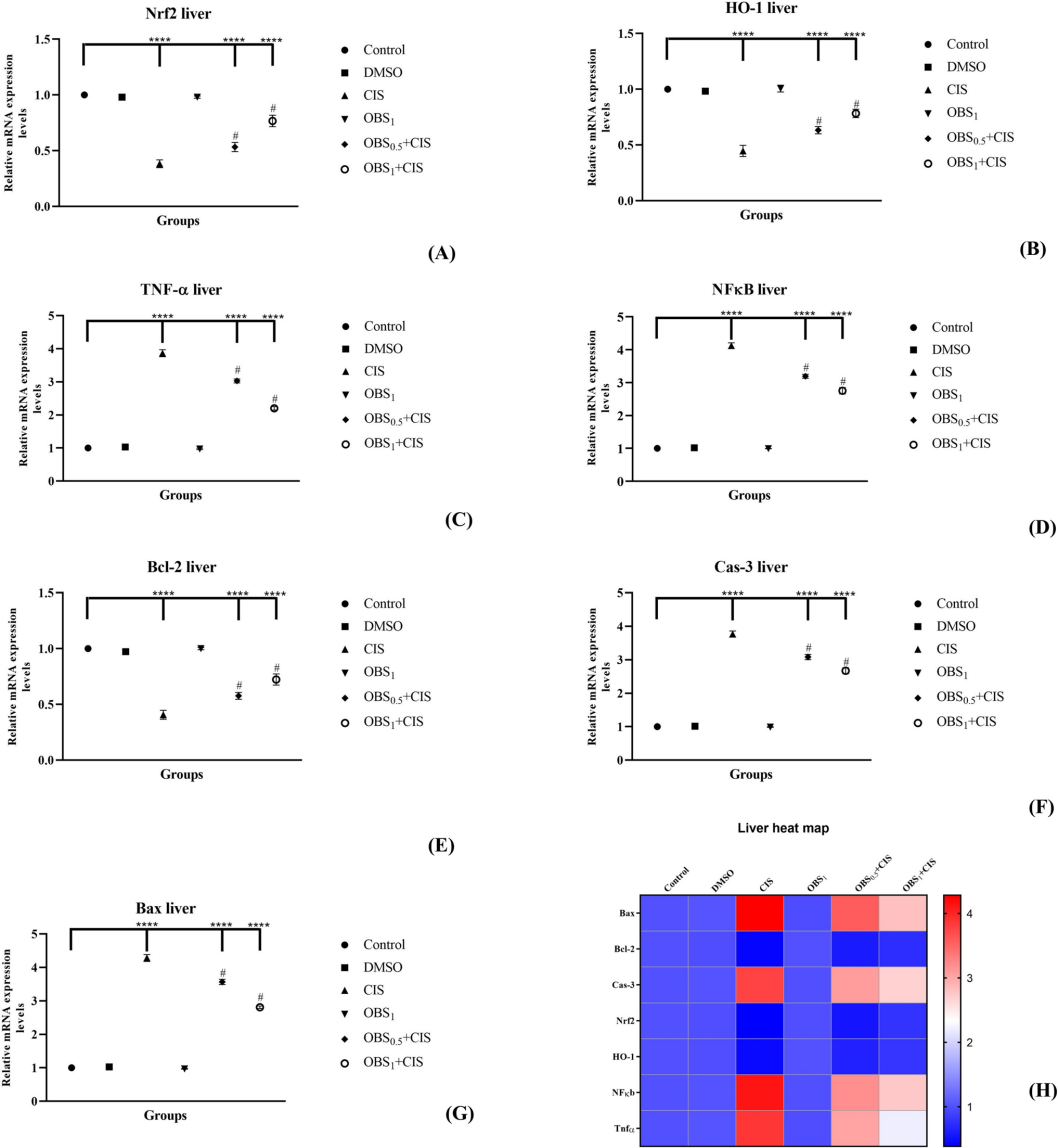
mRNA expression levels of Nrf2 (**A**), HO-1 (**B**), TNF-α (**C**), and NFκB (**D**) which are involved in the Nrf2/HO-1 signaling pathway and Bcl-2 (**E**), Cas-3 (**F**), and Bax (**G**) which are associated with the apoptotic process in liver tissue following obtusifolin (OBS) and cisplatin (CIS) treatments. Corresponding two-dimensional color tone representations of these levels are shown in **H**. Groups: control, DMSO (dimethylsulfoxide, 1%), CIS (20 mg/kg), OBS_1_ (1 mg/kg), OBS_0.5_ + CIS (0.5 mg/kg OBS + 20 mg/kg CIS), and OBS_1_ + CIS (1 mg/kg OBS + 20 mg/kg CIS). Data are presented as mean ± standard deviation; *n* = 7. Values marked with different symbols (*) are statistically significant compared to the control group, while values marked with different symbols (#) are statistically significant compared to the CIS group (*p* < 0.0001)

**Fig. 6:**
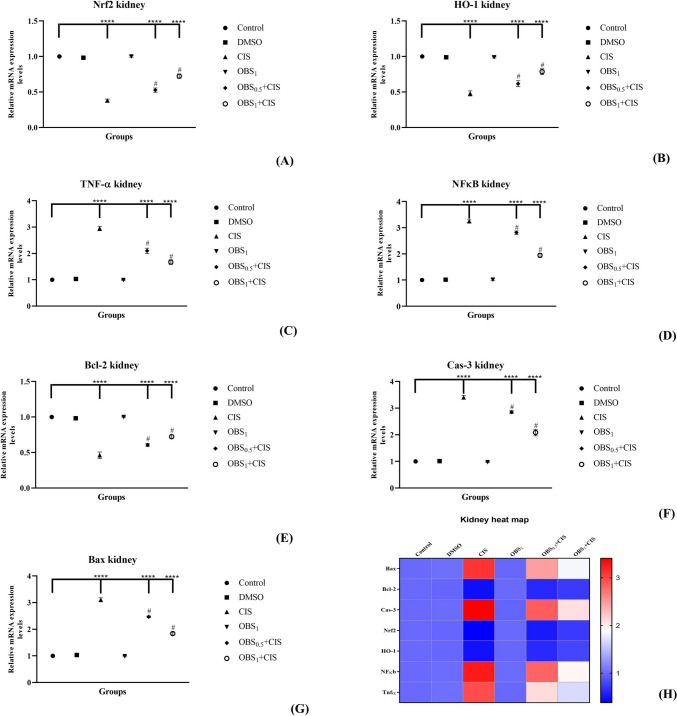
mRNA expression levels of Nrf2 (**A**), HO-1 (**B**), TNF-α (**C**), and NFκB (**D**) involved in the Nrf2/HO-1 signaling pathway and Bcl-2 (**E**), Cas-3, (**F**), and Bax (**G**) associated with the apoptotic process in kidney tissue after obtusifolin (OBS) and cisplatin (CIS) treatments. Corresponding two-dimensional color tone representations are shown in (H). Groups; Control, DMSO (dimethylsulfoxide, 1%), CIS (20 mg/kg), OBS_1_ (1 mg/kg), OBS_0.5_ + CIS (0.5 mg/kg OBS + 20 mg/kg CIS), and OBS_1_ + CIS (1 mg/kg OBS + 20 mg/kg CIS). Data are presented as mean ± standard deviation; *n* = 7. Values marked with different symbols (*) are statistically significant compared to the control group, while values marked with different symbols (#) are statistically significant compared to the CIS group (*p* < 0.0001)

**Fig. 7:**
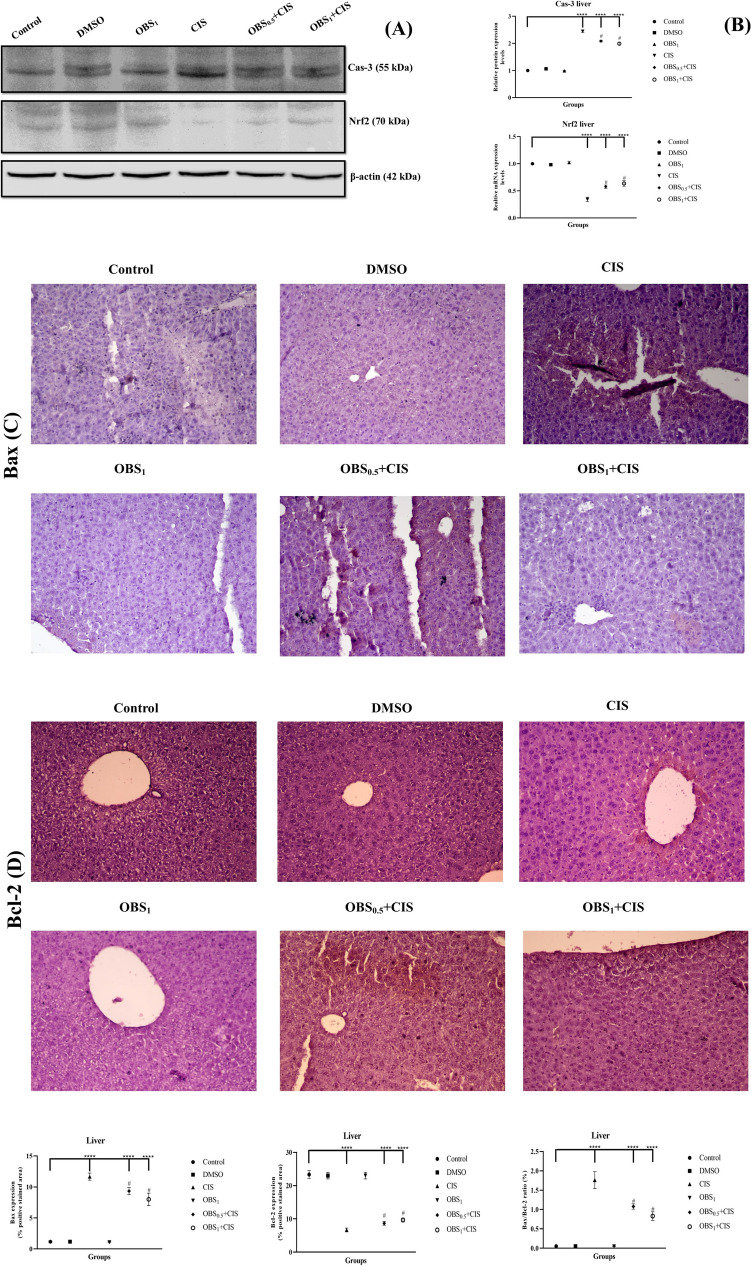
Nrf2 and Cas-3 band images (**A**) with their quantitative analyses (**B**), and Bcl-2 and Bax immunohistochemical staining images (**C**) with corresponding analyses (**D**) in liver tissue following obtusifolin (OBS) and cisplatin (CIS) treatments. Groups; Control, DMSO (dimethylsulfoxide, 1%), CIS (20 mg/kg), OBS_1_ (1 mg/kg), OBS_0.5_ + CIS (0.5 mg/kg OBS + 20 mg/kg CIS), and OBS_1_ + CIS (1 mg/kg OBS + 20 mg/kg CIS). Data are presented as mean ± standard deviation; *n* = 7. Values marked with different symbols (*) are statistically significant compared to the control group, while values marked with different symbols (#) are statistically significant compared to the CIS group (*p* < 0.0001)

**Fig. 8:**
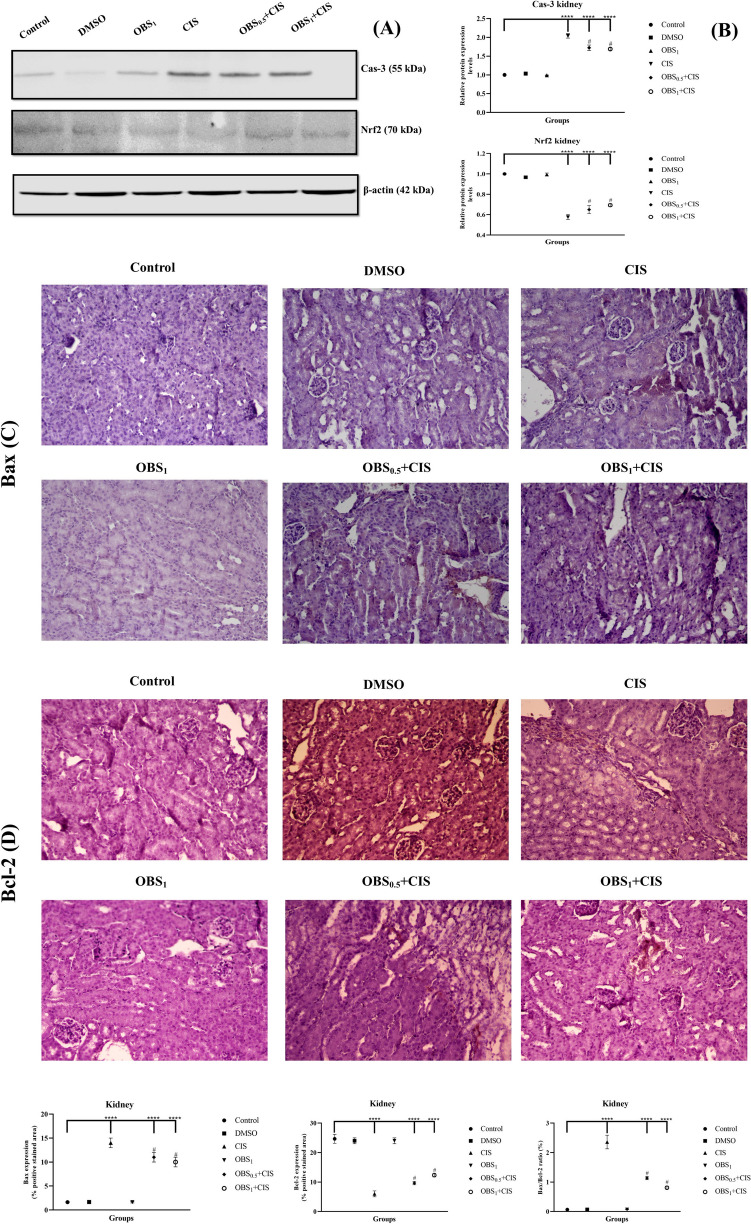
Nrf2 and Cas-3 band images (**A**) with their quantitative analyses (**B**), and Bcl-2 and Bax immunohistochemical staining images (**C**) with corresponding analyses (**D**) in kidney tissue following obtusifolin (OBS) and cisplatin (CIS) treatments. Groups; Control, DMSO (dimethylsulfoxide, 1%), CIS (20 mg/kg), OBS_1_ (1 mg/kg), OBS_0.5_ + CIS (0.5 mg/kg OBS + 20 mg/kg CIS), and OBS_1_ + CIS (1 mg/kg OBS + 20 mg/kg CIS). Data are presented as mean ± standard deviation; *n* = 7. Values marked with different symbols (*) are statistically significant compared to the control group, while values marked with different symbols (#) are statistically significant compared to the CIS group (*p* < 0.0001)

The effects of OBS and CIS treatments on the expression of genes associated with the Nrf2/HO-1 signaling pathway (Nrf2, HO-1, TNF-α, and NFκB) and the apoptotic process (Bcl-2, Cas-3, and Bax) were examined in the liver (Fig. 5) and kidney (Fig. 6) tissues by assessing mRNA and protein expression levels. The results showed that CIS administration led to a decrease in mRNA levels of Nrf2, Bcl-2, and HO-1 while increasing the expression of Cas-3, Bax, NFκB, and TNF-α in both liver and kidney tissues (*p* < 0.0001). On the other hand, OBS increased liver and kidney Nrf2 (by approximately 1.7- to 1.6-fold), HO-1 (by approximately 1.6- to 1.4-fold), and Bcl-2 (by approximately 1.6- to 1.4-fold) mRNA expression levels and suppressed NF-κB (by approximately 0.7–0.7 fold), TNF-α (by approximately 0.6–0.6 fold), Bax (by approximately 0.8- to 0.7-fold), and Cas-3 (by approximately 0.7–0.7 fold) mRNA expression levels compared to CIS group (*p* < 0.0001). These findings were further supported by elevated protein levels (in supplementary material) of Nrf2 (by approximately 1.7- to 1.2-fold) and Bcl-2 (by approximately 1.3- to 1.8-fold) whereas reduced Bax (by approximately 0.7- to 0.8-fold) and Cas-3 (by approximately 0.7–0.7 fold) protein expression in the liver (Fig. 7) and kidney (Fig. 8) tissues (*p* < 0.0001). Notably, OBS alone did not induce any significant changes in these parameters when compared with the control group (*p* > 0.05).

### OBS improves CIS-induced tissue damage

**Fig. 9:**
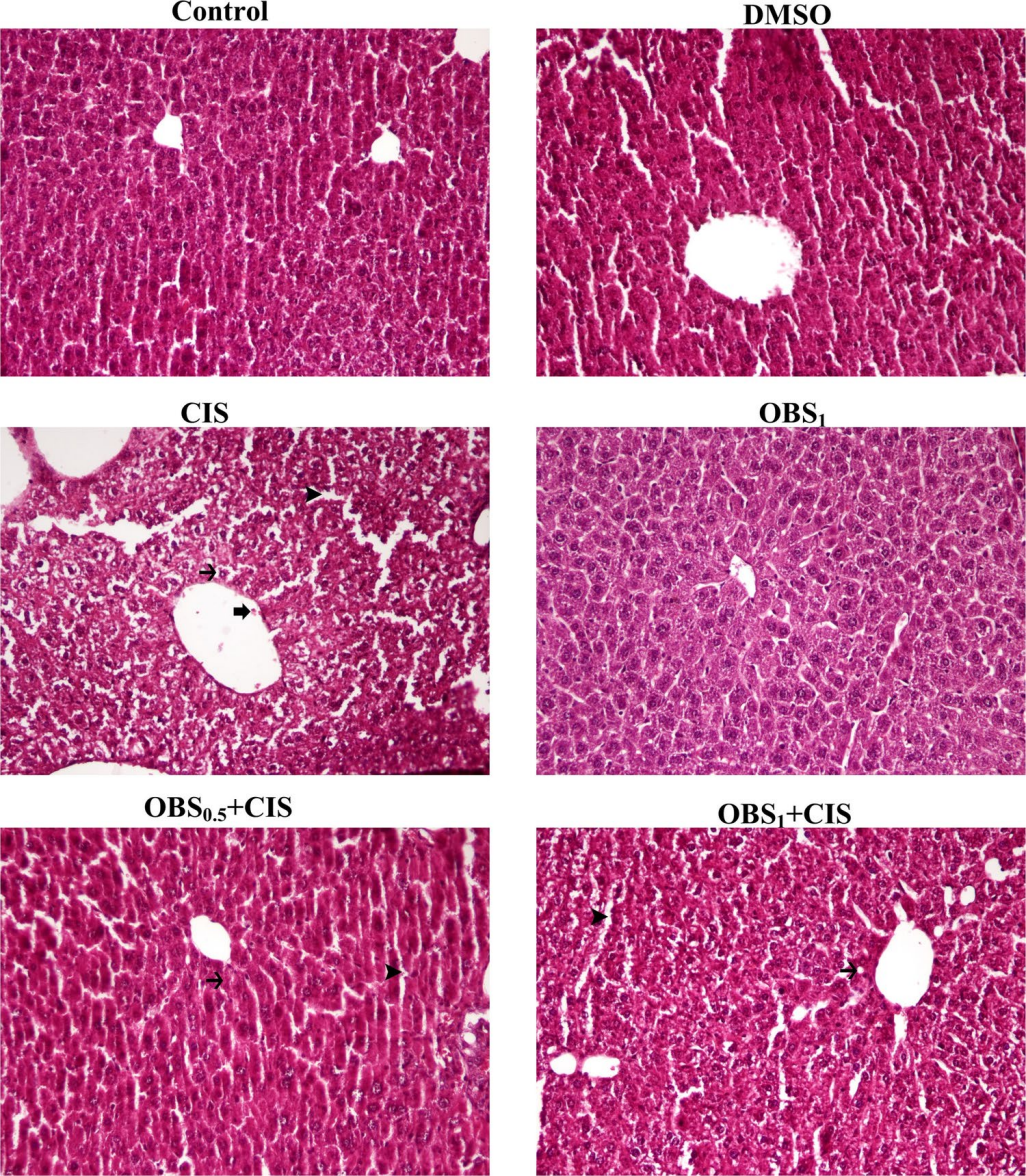
Histopathological changes in liver tissue of mice following obtusifolin (OBS) and cisplatin (CIS) treatments. Sections are stained with H&E (hematoxylin and eosin) and visualized under × 20 magnification (scale bar = 100 µm). Observed pathological features include hyperemia in the central vein (thick arrow), degenerative changes in hepatocytes (thin arrow), and sinusoidal dilation with hyperemia (arrowhead). Groups; Control, DMSO (dimethylsulfoxide, 1%), CIS (20 mg/kg), OBS_1_ (1 mg/kg), OBS_0.5_ + CIS (0.5 mg/kg OBS + 20 mg/kg CIS), and OBS_1_ + CIS (1 mg/kg OBS + 20 mg/kg CIS)

**Fig. 10:**
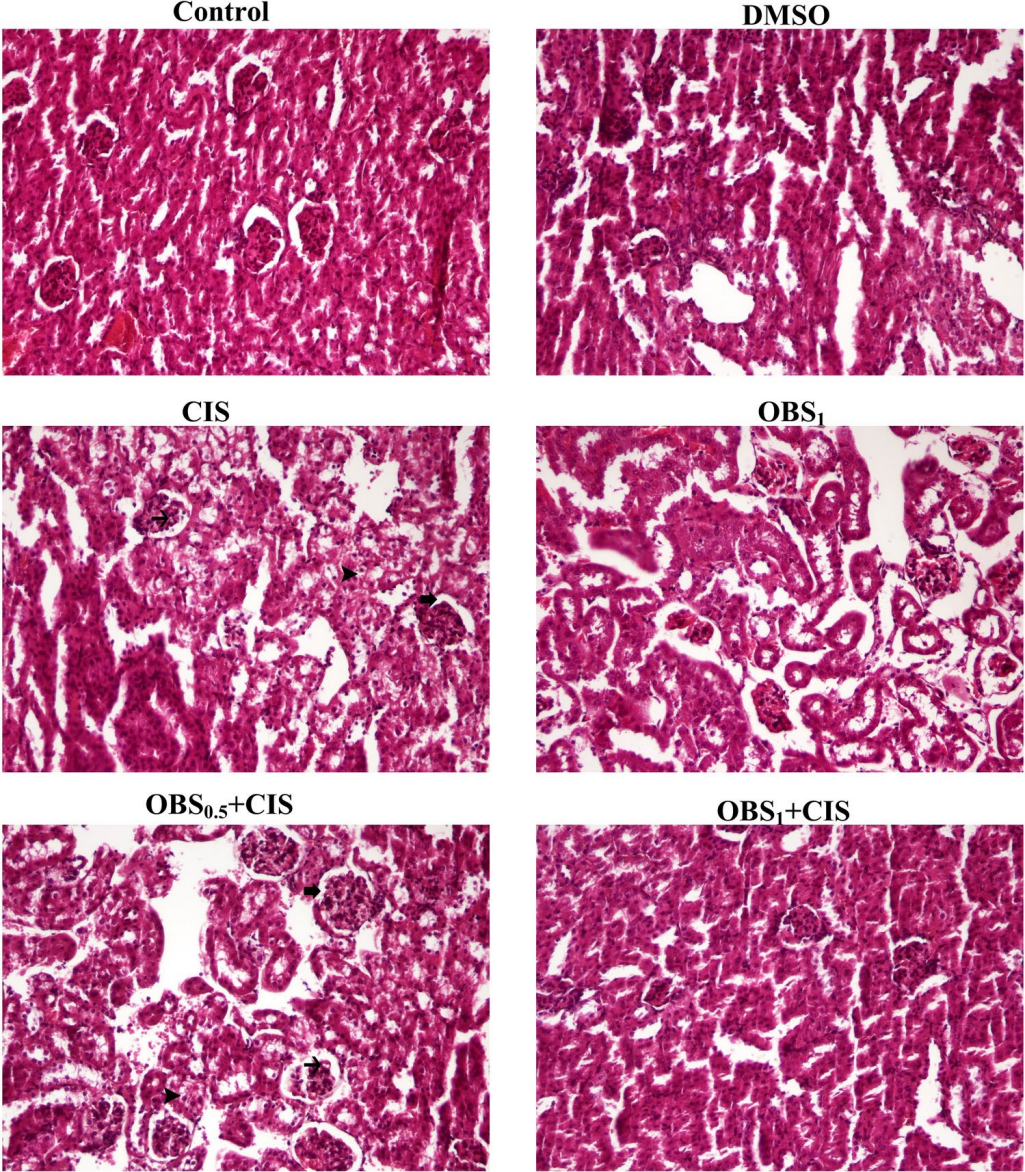
Histopathological changes in kidney tissue of mice following obtusifolin (OBS) and cisplatin (CIS) treatments. Sections are stained with H&E and visualized under × 20 magnification (scale bar = 100 µm). Observed pathological features include Bowman space enlargement (thick arrow) and degenerative changes in the glomerular capillary tuft (thin arrow and arrowhead). Groups; Control, DMSO (dimethylsulfoxide, 1%), CIS (20 mg/kg), OBS_1_ (1 mg/kg), OBS_0.5_ + CIS (0.5 mg/kg OBS + 20 mg/kg CIS), and OBS_1_ + CIS (1 mg/kg OBS + 20 mg/kg CIS)

CIS administration caused marked histopathological changes in the liver, including hyperemia in the central vein, sinusoidal dilation with areas of hyperemia, and degenerative alterations in hepatocytes (Fig. 9). In the kidney, CIS toxicity induced vacuolar degeneration in the glomerular capillary tuft and expansion of Bowman’s space (Fig. 10). However, treatment with OBS significantly mitigated these CIS-induced histopathological damages in both liver and kidney tissues. Furthermore, the group treated with OBS alone showed no histopathological abnormalities compared to the control group. Statistical analyses of the histopathological findings in liver and kidney tissues are presented in Table 2.

**Table 2:** The statistical evaluation of OBS and CIS administration on histopathological alterations in liver and kidney tissue of mice

Tissue	Histopathological alterations	Groups					
		Control	DMSO	CIS	OBS_1_	OBS_0.5_ + CIS	OBS_1_ + CIS
Liver	Hyperemia in the central vein	0.00 ± 0.00^c^	0.00 ± 0.00^c^	2.28 ± 0.48^a^	0.00 ± 0.00^c^	1.85 ± 0.69^ab^	1.42 ± 0.53^b^
	Degenerative changes in hepatocytes	0.00 ± 0.00^c^	0.00 ± 0.00^c^	2.57 ± 0.54^a^	0.00 ± 0.00^c^	2.14 ± 0.89^ab^	1.57 ± 0.53^b^
	Sinusoidal dilatation and hyperemia	0.00 ± 0.00^c^	0.00 ± 0.00^c^	2.42 ± 0.53^a^	0.00 ± 0.00^c^	2.14 ± 0.69^ab^	1.42 ± 0.97^b^
Kidney	Enlargement of the Bowman’s space	0.00 ± 0.00^c^	0.00 ± 0.00^c^	2.85 ± 0.37^a^	0.00 ± 0.00^c^	2.42 ± 0.53^ab^	1.71 ± 0.95^b^
	Vacuolar degeneration in the glomerular capillary tuft	0.00 ± 0.00^c^	0.00 ± 0.00^c^	2.28 ± 0.48^a^	0.00 ± 0.00^c^	1.85 ± 0.69^ab^	1.14 ± 0.69^b^

Mean ± standard deviations; *n*: 7; values with different letters in the same row are statistically significant (*p* < 0.05)

Abbreviations: *OBS*, obtusifolin; *CIS*, cisplatin

## Discussion

Hepatotoxicity and nephrotoxicity remain significant complications with a high prevalence among patients undergoing CIS treatment, prompting numerous clinical and experimental studies to address these challenges (Ince et al. 2014; Elhady et al. 2022). The present study aimed to evaluate the protective effects of OBS against CIS-induced hepatotoxicity and nephrotoxicity in mice.

CIS administration caused considerable liver and kidney damage, as evidenced by elevated serum levels of BUN, AST, ALT, ALP, and creatinine, which are key biomarkers of hepatotoxicity and nephrotoxicity (Elhady et al. 2022). In this study, liver and kidney enzyme levels were significantly increased following CIS administration. However, treatment with OBS effectively mitigated these elevations, highlighting its hepatonephroprotective properties. Consistent with these findings, Xiang et al. (2024) reported that OBS ameliorated acute kidney injury by reducing BUN and creatinine levels induced by lipopolysaccharide. Similarly, an extract from *Cassia obtusifolia* seeds, a source of OBS, demonstrated hepatoprotective effects by reducing elevated ALT and AST levels in carbon tetrachloride-induced hepatic injury (Xie et al. 2012).

CIS stimulates the generation of reactive oxygen species (ROS), exacerbating oxidative stress by depleting non-enzymatic antioxidants like GSH and enzymatic antioxidants such as SOD and CAT, ultimately leading to toxicity (Abdel-Daim et al. 2020). Consistent with previous research (Abo-Elmaaty et al. 2020; Habib et al. 2021), this study demonstrated that CIS administration in mice caused a significant increase in MDA levels in both the liver and kidneys, accompanied by a substantial reduction in GSH levels as well as SOD and CAT activities compared to the control group. Moreover, a study on diabetic animals found that OBS treatment led to a dose-dependent decrease in MDA levels, along with increases in GSH levels and SOD and CAT activities in treated diabetic groups compared to untreated groups. This study concluded that OBS exhibits antioxidant properties, mitigating oxidative stress and improving diabetes and its complications (Tang and Zhong 2014). Similarly, Zhuang et al. (2016) reported that intragastric administration of OBS (5 and 20 mg/kg) for four weeks increased SOD and NO levels while reducing serum MDA levels in hyperlipidemic rats induced by a high-fat diet. In alignment with these findings, the current study demonstrated that dose-dependent OBS treatments reduced CIS-induced MDA levels while restoring GSH levels and enhancing SOD and CAT activities. These results confirm that OBS possesses potent antioxidant properties, effectively reducing oxidative stress in tissues.

Nrf2 plays a critical role in regulating the expression of numerous antioxidant genes (such as HO-1, SOD, CAT, and GPx) and pro-inflammatory genes (including NFκB, TNF-α, and IL-1β) (El-Sayed et al. 2021; Ince et al. 2024). Research has demonstrated that pharmacologically active compounds capable of activating Nrf2 can effectively reduce oxidative stress and inflammation, thereby mitigating liver and kidney damage caused by chemotherapeutic agents (Alanezi et al. 2022; Tureyen et al. 2023). As such, Nrf2-activating compounds offer significant therapeutic potential against CIS-induced hepatonephrotoxicity. In this study, OBS significantly upregulated HO-1 and Nrf2 expression in the livers and kidneys of mice exposed to CIS toxicity. These findings align with previous research emphasizing the central role of Nrf2 in mediating the pharmacological effects of OBS. For example, Ren et al. (2023) reported that OBS exerted its pharmacological effects through Nrf2-EpRE activation in CALUX cells. In the livers and kidneys of CIS-treated mice, OBS suppressed NFκB activity and reduced TNF-α levels, demonstrating its potent anti-inflammatory properties. Additionally, OBS alleviated CIS-induced apoptosis by decreasing Bax and Cas-3 expression while upregulating Bcl-2. The anti-inflammatory effects of OBS have also been confirmed in a rat model of lipopolysaccharide-induced kidney injury, where it inhibited the NFκB signaling pathway and reduced elevated Bax and Cas-3 levels (Xiang et al. 2024). Furthermore, OBS has been shown to regulate MUC5AC mucus mRNA expression and synthesis in airway epithelial cells by modulating the NFκB pathway (Choi et al. 2019), and to reduce inflammation in an osteoarthritis model via NFκB signaling inhibition (Nam et al. 2021). Thus, the protective effects of OBS against CIS-induced oxidative stress and inflammation in the liver and kidneys are likely attributable to its modulation of the Nrf2/HO-1 pathway and its inhibition of the NFκB signaling pathway.

CIS administration resulted in histopathological alterations in liver tissues, primarily characterized by hyperemia and degenerative changes, and in kidney tissues, marked by vacuolar degeneration and expansion of Bowman’s space. These findings align with previous studies reporting that CIS induces histopathological lesions in the kidney and liver tissues of treated mice (Folayan et al. 2022; Elhady et al. 2022; Li et al. 2023). Conversely, OBS treatment demonstrated a protective effect by mitigating CIS-induced cellular damage in these tissues. This observation is further supported by previous research showing that OBS prevents microvascular apoptosis and pathological changes in the retina of diabetic models (Hou et al. 2014).

This study is the first to evaluate OBS in a therapeutic context against CIS toxicity. One of the strengths of our study was the evaluation of the protective effects of OBS on liver and kidney toxicity. In addition, intraperitoneal administration of OBS allowed for better absorption, allowing for more precise experimental control. Although our study showed promising preclinical preventive results, it has limitations such as not evaluating its clinical therapeutic efficacy and a need to assess the ratio of pro-caspase-3 to cleaved caspase-3 and Nrf2 in cytosolic and nuclear fractions, which was our primary focus. Future studies should investigate these aspects of OBS to understand its potential fully.

## Conclusion

Our study demonstrated that OBS administration effectively mitigated CIS-induced inflammation, oxidative stress, and cell death in the kidney and liver tissues of mice, primarily through the activation of the Nrf2/HO-1 pathway. These findings suggest that similar to other anthraquinone derivatives, OBS holds promise as a potential candidate for the development of novel hepatonephroprotective adjuvants or therapeutic agents.

## Supplementary Information

Below is the link to the electronic supplementary material.

Supplementary file1 (DOCX 867 KB)

## Data Availability

All data generated or analyzed during this study are included in the manuscript.
