# Supplementary material for: Obtusifolin improves cisplatin-induced hepatonephrotoxicity via the Nrf2/HO-1 signaling pathway
Source: Naunyn Schmiedebergs Arch Pharmacol. 2025 Feb 20;398(8):10337–52. doi: 10.1007/s00210-025-03900-x (PMC12350600; doi:10.1007/s00210-025-03900-x)

**Supplementary data**

**Figure 6.** Original Western blot images

**Liver Cas-3 (55-kDa)**

| Control | DMSO | OBS_1_ | CIS | OBS_0.5_+CIS | OBS_1_+CIS |
| --- | --- | --- | --- | --- | --- |


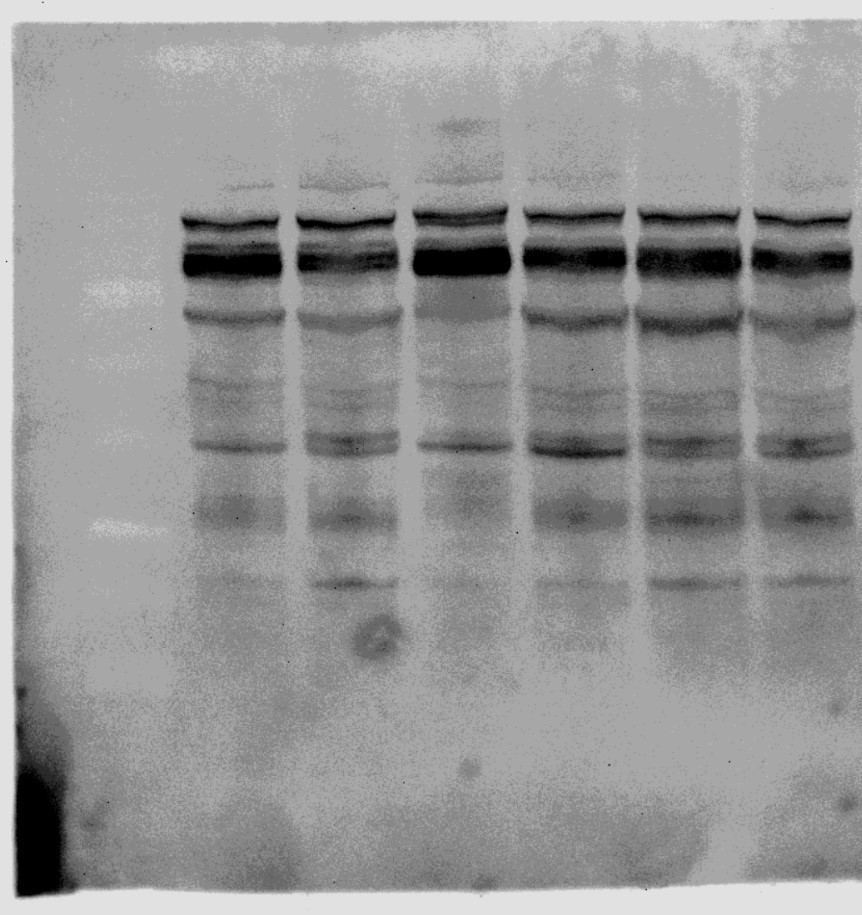


**Liver Nrf2 (70kDa)**

| Control | DMSO | OBS_1_ | CIS | OBS_0.5_+CIS | OBS_1_+CIS |
| --- | --- | --- | --- | --- | --- |


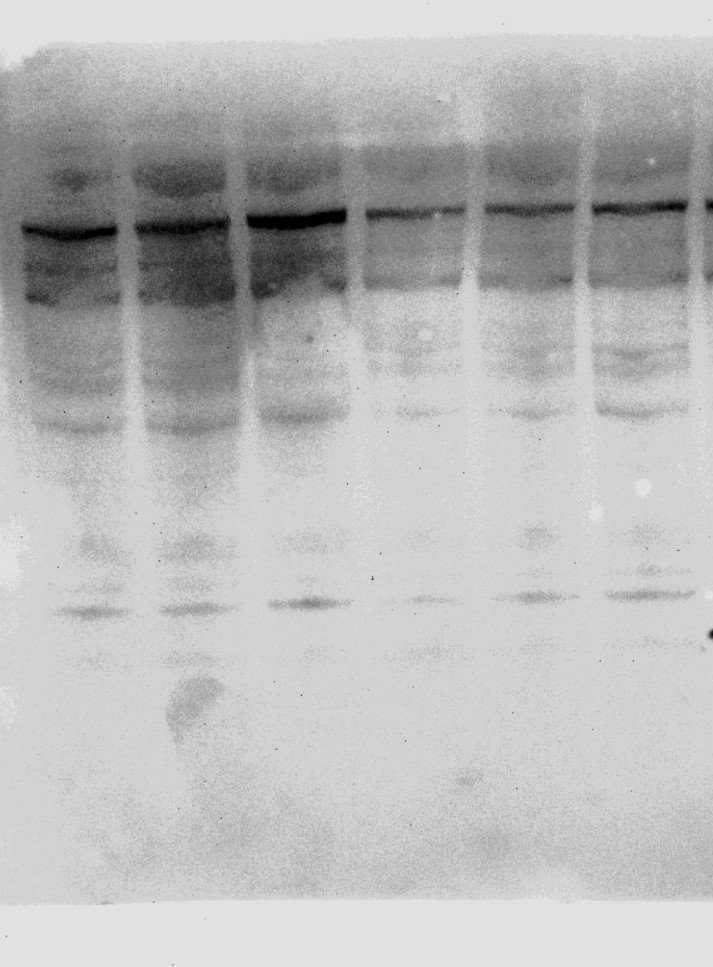


**Liver β-actin (42 kDa)**

| Control | DMSO | OBS_1_ | CIS | OBS_0.5_+CIS | OBS_1_+CIS |
| --- | --- | --- | --- | --- | --- |


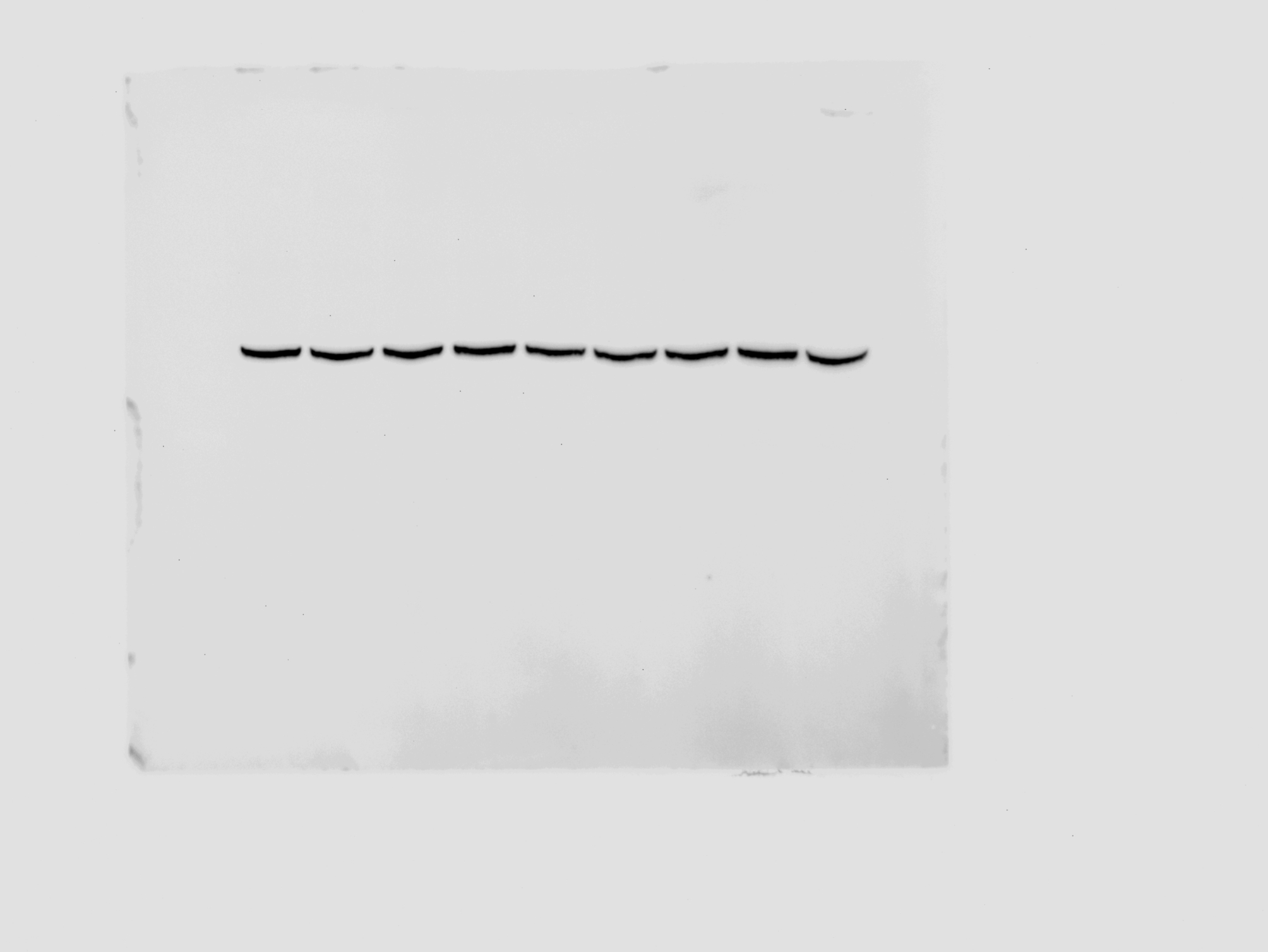


**Figure 7.** Original Western blot images

**Kidney Cas-3 (55-kDa)**

| Control | DMSO | OBS_1_ | CIS | OBS_0.5_+CIS | OBS_1_+CIS |
| --- | --- | --- | --- | --- | --- |


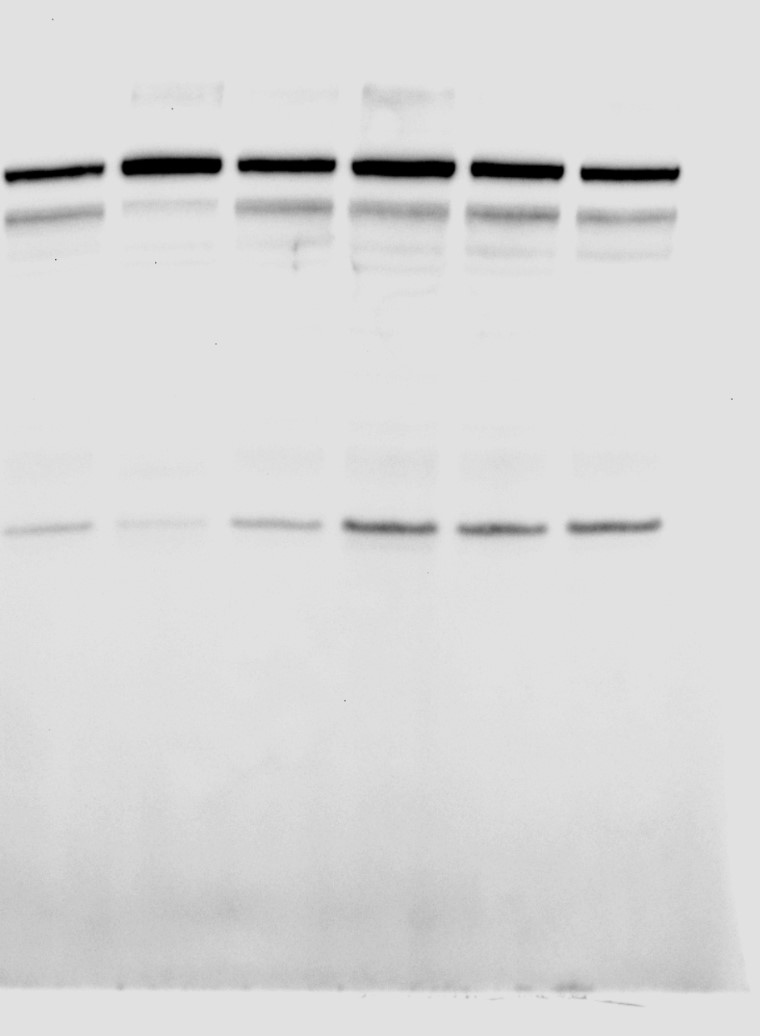


**Kidney Nrf2 (70kDa)**

| Control | DMSO | OBS_1_ | CIS | OBS_0.5_+CIS | OBS_1_+CIS |
| --- | --- | --- | --- | --- | --- |


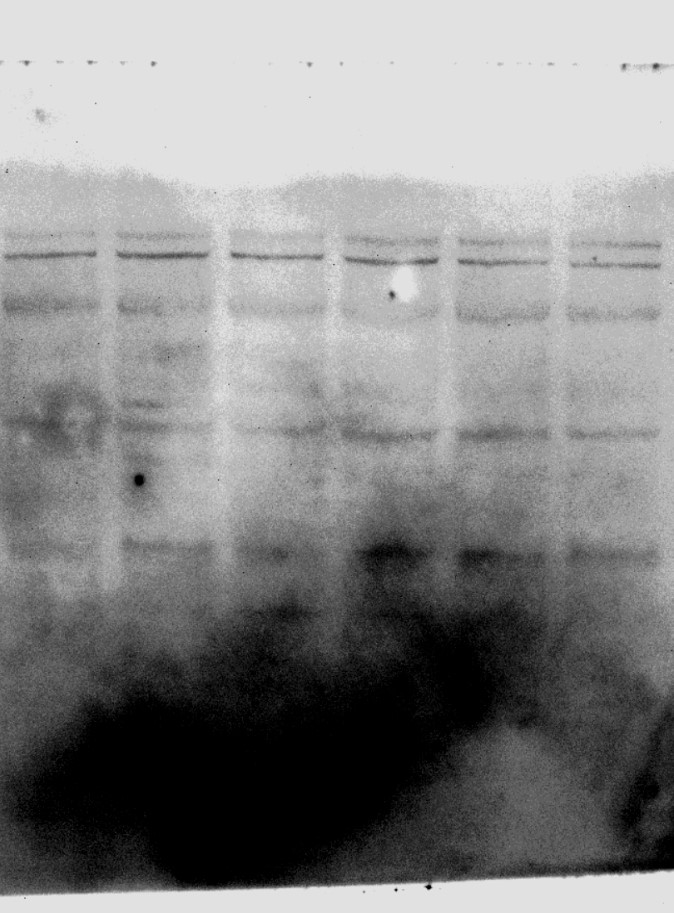


**Kidney β-actin (42 kDa)**

| Control | DMSO | OBS_1_ | CIS | OBS_0.5_+CIS | OBS_1_+CIS |
| --- | --- | --- | --- | --- | --- |


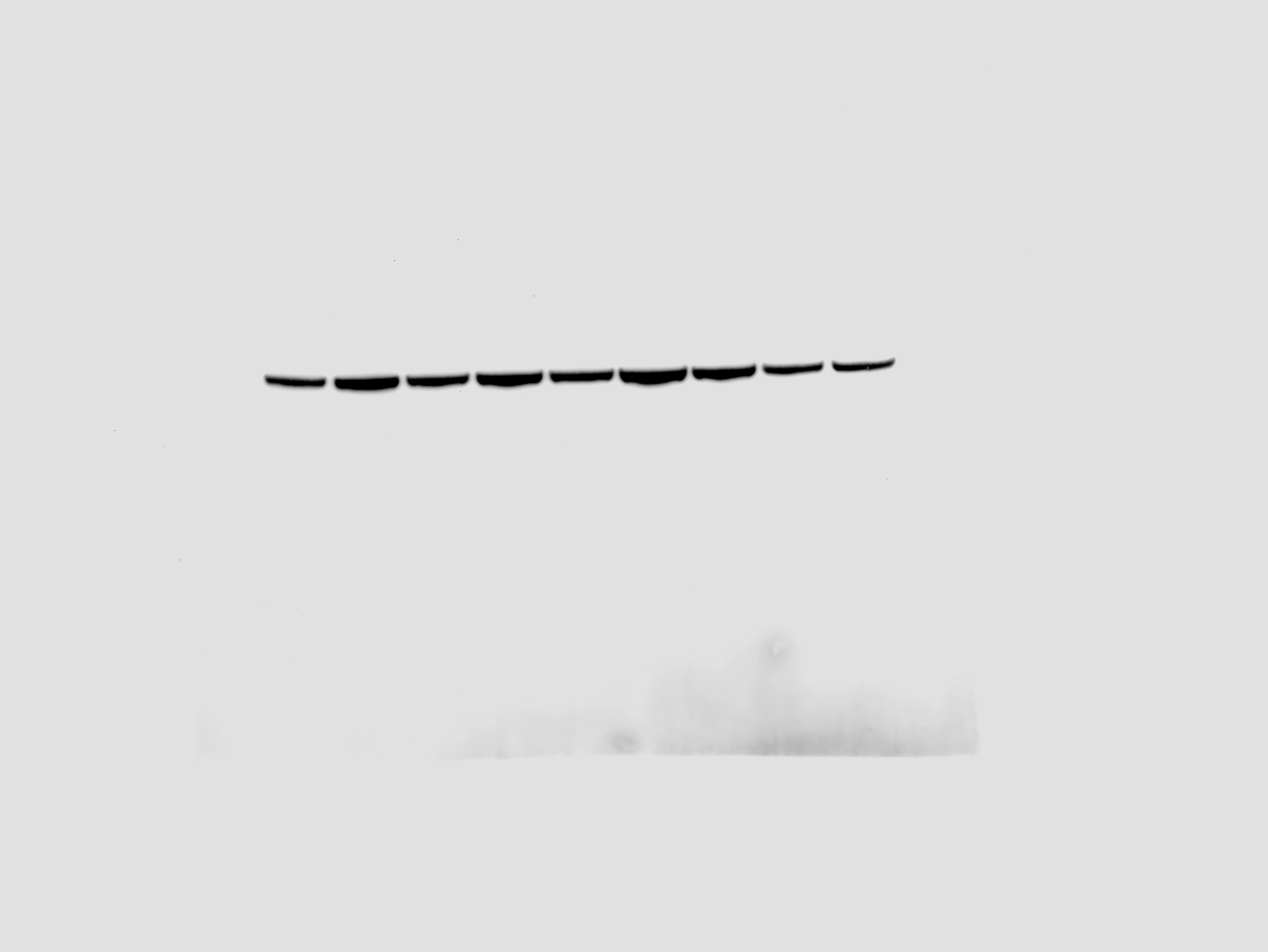

Supplement: Supplementary file 1 — Supplementary file1 (DOCX 867 KB) [file 210_2025_3900_MOESM1_ESM.docx]
